# DNA Aptamer Beacon Probe (ABP) for Monitoring of Adenosine Triphosphate Level in SW480 Cancer Cells Treated with Glycolysis Inhibitor 2-Deoxyglucose

**DOI:** 10.3390/ijms24119295

**Published:** 2023-05-26

**Authors:** Katarzyna Ratajczak, Magdalena Stobiecka

**Affiliations:** Department of Physics and Biophysics, Warsaw University of Life Sciences, 159 Nowoursynowska Street, 02776 Warsaw, Poland

**Keywords:** aptamer beacon probe (aptaprobe, ABP), ATP detection, biosensing assay, fluorescence spectroscopy, SW480 cancer cells, 2-deoxyglucose (2-DG) glycolysis inhibitor

## Abstract

Early cancer screening enables timely detection of carcinogenesis, and aids in prompt clinical intervention. Herein, we report on the development of a simple, sensitive, and rapid fluorometric assay based on the aptamer probe (aptamer beacon probe, ABP) for monitoring the energy-demand biomarker adenosine triphosphate (ATP), an essential energy source that is released into the tumor microenvironment. Its level plays a significant role in risk assessment of malignancies. The operation of the ABP for ATP was examined using solutions of ATP and other nucleotides (UTP, GTP, CTP), followed by monitoring of ATP production in SW480 cancer cells. Then, the effect of a glycolysis inhibitor, 2-deoxyglucose (2-DG), on SW480 cells was investigated. The stability of predominant ABP conformations in the temperature range of 23–91 °C and the effects of temperature on ABP interactions with ATP, UTP, GTP, and CTP were evaluated based on quenching efficiencies (QE) and Stern-Volmer constants (*K*_SV_). The optimized temperature for best selectivity of ABP toward ATP was 40 °C (*K*_SV_ = 1093 M^−1^, QE = 42%). We have found that the inhibition of glycolysis in SW480 cancer cells by 2-deoxyglucose resulted in lowering of ATP production by 31.7%. Therefore, monitoring and modulation of ATP concentration may aid in future cancer treatment.

## 1. Introduction

In view of the rising numbers of cancer cases around the world, extensive efforts have recently been devoted to developing novel effective cancer treatments, addressing specific features of carcinogenesis, tumor growth, and metastasis. Cancer is currently the leading cause of death worldwide, with 19.3 million new cases and near 10 million deaths reported in 2020 [1]. Colorectal cancer (CRC) is the third-most common malignancy and the second-most deadly cancer type worldwide. The global number of new CRC cases (colon, rectal, anal) is predicted to reach 3.2 million in 2040, based on projections of aging, population growth, and human development [2]. It is estimated that over 153,000 new CRC cases will be diagnosed in the United States in 2023 [3]. 

Due to fast tumor growth, invasion into neighboring cells, and cancer cells’ migration, early diagnosis and prompt treatment are crucial in the fight against cancer [4]. The timely detection of cancer biomarkers, even before symptoms appear, plays a pivotal role in successful cancer treatment. To achieve this end, a widespread cancer screening has been advised. Due to the large number of subjects involved in such a cancer screening, the methods used for cancer biomarker detection must fulfill the requirements of low cost, simple operation, and high sensitivity and reliability. Therefore, it is of great urgency to develop biosensing probes for prognostic cancer biomarker detection in early cancer screening. In previous studies [5,6,7], we have designed highly sensitive biosensing systems for the detection of cancer biomarkers, including the anti-apoptotic protein survivin and its mRNA. The proposed systems were based on fluorescent molecular-beacon probes, and were applied for testing SW480 human colorectal cancer cells. We have also designed an immunosensor based on monoclonal antibodies against protein survivin [8]. In the present work, we have focused on the development of a new biosensing platform for monitoring adenosine triphosphate (ATP), whose production is upregulated in cancer cells to support the elevated demands for energy supply needed by fast-growing and proliferating cancer cells. 

The adenosine triphosphate plays a key role in many biologically important processes [9], such as proliferation [10], metabolism [11,12], differentiation [13] and apoptosis [14], where the main function of ATP is to provide energy to cellular processes, including facilitating energy transfer, intracellular signalling, and the synthesis of proteins, DNA, and RNA [15]. 

Recently, it has been found that the ATP level is also increased in the tumor microenvironment (TME). The ATP is released into the TME by the tumor itself, as well as by the host cells, and can be involved in the migration and activation of immune cells, in addition to supporting the metabolism and growth of tumor and stromal cells. Thus, TME ATP is often considered to be a biochemical hallmark of the host–tumor interface [16,17,18]. Yang et al. [19,20] have shown that extracellular ATP can promote breast carcinoma invasion, and plays a vital role in mediating breast cancer chemoresistance. Salvestrini et al. [14] have discovered that the activation of ATP plasma membrane ion channels (P2X7R) with high doses of ATP can induce acute apoptosis of myeloid leukemia (AML) blast cells. Fiorillo et al. [15,21] have found that the ATP-high MDA-MB-231 cells exhibit a 20–40-fold and 15–60-fold increase in their ability to undergo cell migration and invasion, respectively, relative to the ATP-low cell population. Furthermore, it appears that in the drug-resistant cells, mitochondrial ATP production is dysregulated, leading to enhanced aerobic glycolysis and elevated intracellular ATP levels, as well as increased levels of hypoxia-inducible factor-1 alpha (HIF-1α) [22].

Therefore, due to its active role in carcinogenesis, ATP may be considered a non-specific cancer biomarker that may become a new suitable molecular target in cancer therapy, enabling the hinderance of metastasis and cancer propagation. In addition, the determination of the intracellular ATP concentration in biological samples is crucial for monitoring of disease progression. 

It has generally been accepted that one of the hallmarks of neoplastic diseases is the change in energy metabolism, with an upsurge in glycolysis, producing energy-carrying ATP molecules, at the cost of the usual oxidative phosphorylation (OXPHOS), even under normoxic conditions. This change, known as the Warburg effect, provides a vast amount of energy that cancer cells need for extensive proliferation [23,24,25]. The modulation of ATP production in cancer cells by glycolysis inhibitors may become a novel approach to control cancer growth and proliferation. Martin et al. [26,27] have reported that cancer treatment in combination with ATP-depleting agents and anticancer drugs markedly enhanced tumor regression, e.g., MAP = 6-methylmercaptopurine riboside (MMPR) + 6-aminonicotinamide (6-AN) + *N*-(phosphonacetyl)-L-aspartic acid (PALA) with two anticancer agents: 5-fluorouracil (FU) + Adriamycin (Adr) [28], MAP + FU [29]. Wang et al. [30] have also shown that the depletion of ATP can be considered a therapeutic strategy for cancer treatment. The authors have utilized micelles composed of branched polyethylene glycol (bPEG), Pluronic P123 cationic multiblock copolymer, and low molecular weight polyethyleneimine (PEI) (PSPP/PTX/siRNA) as a codelivery carrier of small interfering RNA (siRNA) against polo-like kinase1 (PLK1) and paclitaxel (PTX) for effective reversal of cancer multidrug resistance (MDR) in vitro and in vivo. Song et al. [31] have applied a smart nanoagent based on the assembly of quantum dots (QDs), tannic acid (TA), and doxorubicin (DOX) chemodrug (QDs@TA-PEG/DOX) for depleting intracellular ATP content and increasing cells’ chemosensitivity. 

The decrease of intracellular ATP production by treating cancer cells with 2-deoxyglucose (2-DG) was recently reported by Kaushik et al. [32], who applied luciferase and luciferin-based assay and observed a 12% and 16% intracellular ATP level decrease in THP-1 and U937 human leukemic cancer cell lines, respectively, when treated for 24 h with 1 mM 2-DG. The treatment of human lung A549 carcinoma cells with 20 mM 2-DG for 2.5 h also resulted in ca. 30% ATP level suppression [33]. According to Maximchik et al. [34], the treatment with 20 mM 2-DG caused about a 25–30% drop in the ATP level in HCT116 human colorectal carcinoma cell line, and a 60–80% ATP level drop in the SK-N-BE(2) neuroblastoma cell line. Sahra et al. [35] have observed a 60% decrease of intracellular ATP concentration in LNCaP androgen-sensitive human prostate adenocarcinoma cells treated for 24 h with 1 mM 2-DG. 

The results presented above indicate that the excess energy production associated with cancer progression can be restrained by applying inhibitors of glycolysis. These inhibitors suppress the intracellular ATP level and contribute to the retardation of tumor growth and metastasis.

In studies of processes leading to the suppression of energy supply, the measurement of the level of ATP, the main energy source, becomes the key factor for successful assessment of the progress achieved. Therefore, together with the monitoring of biomarkers specific to various cancer types, the monitoring of ATP is one of the most important issues. Although a range of assays and biosensing platforms for ATP determination have been proposed, a highly sensitive, inexpensive, and rapid method is desired for possible application in widespread cancer screening. Among these methods, some of the most promising are listed below. 

Recently, several methods for the detection of ATP, based on aptamers, have been proposed. The aptamers used included synthetic DNA or RNA oligonucleotides that bind with high affinity and specificity to the targeted molecules [36,37,38]. The first successfully selected DNA aptamer that binds adenosine and ATP in solutions was outlined in 1995 by Huizenga and Szostak [39]. This aptaprobe, which consisted of two small Watson-Crick helices and two G-quartets, is still one of the most-studied aptamers for ATP detection. However, some changes and modifications of these aptamers have recently been proposed and utilized in more complex biosensing systems and biosensors to achieve a lower limit of detection for ATP, at the cost of simplicity and rapidity of the assay operation [40,41,42,43]. The complex assay developed by Guo [44], based on two aptamers and a multiplication circle, was able to achieve a remarkably low limit of detection for ATP: 38 nM. Zhou et al. [45] have developed a platform based on surface-enhanced Raman spectroscopy (SERS). The authors have utilized split aptamers (apt1 and apt2) attached to a nanolayer of gold nanoparticle-decorated graphene oxide (GO/AuNP) and gold nanoparticles (AuNP), respectively. In the presence of ATP, interactions with split aptamers on AuNP have occurred, leading to a closer proximity between nanomaterials. This results in enhanced SERS signals for the detection of ATP, and a very low limit of detection: LOD = 0.85 pM ATP.

Due to the complex nature of neoplasia, associated with the effects of multiple factors such as genes, proteins, and biologic pathways, it is necessary to develop analytical biosensing platforms based on different panels, specific to different relevant biomarkers. Such a multi-panel platform can offer a high reliability for cancer diagnostics and screening. Recently, growing evidence indicates that the use of panels of biomarkers offers a significant improvement in cancer diagnostic accuracy [46,47,48]. 

In our previous studies, we have developed biosensing systems based on molecular beacons and biosensors for the detection of survivin mRNA and other cancer biomarkers [4,5,6]. We have also shown that the fluorescein modified ATP aptamer may be used for monitoring ATP levels in untreated and oligomycin-treated cancer cells [49]. 

**Scheme 1:**
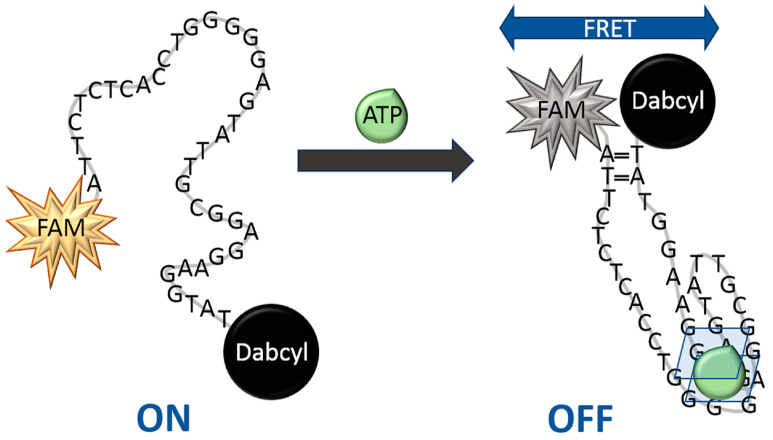
Schematic view of the conversion of the aptamer beacon probe (ABP) from the random coil conformation to the G-quadruplex in the presence of ATP. The random coil ABP is in equilibrium with predominant structures 1–4, presented in Figure 1.

In the present work, we have developed a novel aptamer beacon probe (aptaprobe, ABP) modified with fluorescein (FAM) at the 5′ end, and quencher (Dabcyl) at the 3′ end of the DNA oligonucleotide with G-quadruplex structure. The new aptamer beacon probe consists of a single-stranded oligonucleotide loop and a duplex stem, formed by self-complementary ends of the strand containing two bases, thus resembling a specific hairpin structure. The sequence of ABP was based on the DNA strand originally developed by Huizenga and Szostak [39] that involved the G-rich 27-nucleotide sequence of ATP-binding aptamer 5′-ACCTGGGGGAGTATTGCGGAGGAAGGT-3′. This strand was extended in the present work with the addition of seven nucleotides—ATTCTCTC—at the 5′ end, and two nucleotides—AT—at the 3′ end of this sequence. Since the loop of ABP is rich in guanines, during the interactions with ATP, the square planar structure of the characteristic G-quadruplex can be formed (Scheme 1). Upon binding of ATP to ABP, the FAM fluorescent dye and the Dabcyl quencher, which are attached at the ends of ABP sequence and are widely separated from each other, get closer together, enabling an efficient Forster resonance energy transfer (FRET), resulting in FAM fluorescence quenching, as depicted in Scheme 1. First, we have performed physicochemical investigations of this aptamer probe in buffer solutions. Our initial aim was to evaluate the effects of several experimental parameters including the incubation time of aptaprobe and the experimental temperature on biosensing assay performance. The ATP aptamer selectivity against other nucleoside triphosphates such as GTP, CTP, and UTP was also evaluated on the basis of quenching efficiencies (QE) and Stern-Volmer constants (*K*_SV_). Furthermore, testing of the assay developed using biological samples was performed. The SW480 human colorectal cancer cells lysate (300-fold diluted) and lysate from SW480 cells treated with 2-deoxyglucose glycolysis inhibitor (300-fold diluted) were used for this purpose. We have demonstrated that the proposed simple and rapid fluorometric assay based on DNA aptamer beacon can be designed and used for the detection and monitoring of intracellular ATP levels in cancer cells for diagnostics and prognostication. The obtained results also indicate that further investigation is warranted to follow the modulation of ATP levels by glycolysis inhibitors as a potential cancer treatment.

## 2. Results and Discussion

### 2.1. Characterization of the ATP Aptamer Beacon Probe 

**Figure 1:**
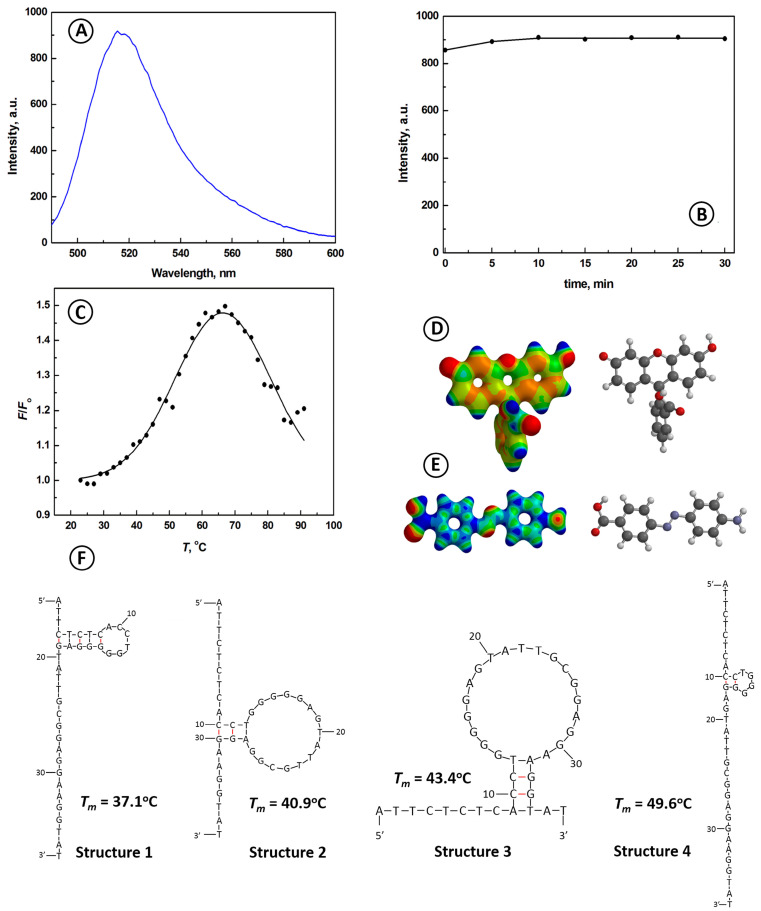
(**A**) Fluorescence emission spectrum for aptamer beacon probe (aptaprobe, ABP) for *λ*_ex_ = 480 nm; (**B**) Dependence of the fluorescence emission intensity of aptaprobe on time for peak emission at *λ*_em_ = 517 nm; (**C**) The melting characteristics for aptaprobe, *λ*_em_ = 517 nm; (**D**,**E**) Electronic and chemical structures of the fluorophore fluorescein; (**D**) Quencher Dabcyl (**E**) attached to the ends of the aptaprobe nucleotide sequence; electron density surfaces (d = 0.08 a.u.) with mapped electrostatic potential (color coded: from negative-red to positive—blue); (**F**) main theoretical aptaprobe structures with their melting temperatures. Conditions: *C*_ABP_ = 133 nM, *λ*_ex_ = 480 nm, buffer solution: 20 mM Tris-HCl + 100 mM NaCl + 5 mM MgCl_2_, room temperature 21 °C.

The aptamer beacon probe (ABP) designed in this work consists of a single-stranded 37-mer oligonucleotide, with the strand sequence: 5′-ATTCTCTCACCTGGGGGAGTATTGCGGAGGAAGGTAT-3′, and a fluorophore-quencher pair attached to the ends of the strand. The fluorophore (fluorescein, FAM) of the pair was bonded to the 5′ end of the oligonucleotide strand, and the quencher (Dabcyl) was bonded to the 3′ end of the strand. The electronic and chemical structures of FAM and Dabcyl are presented in Figure 1 in panels D–E, with the electrostatic potential mapped on the constant electron density surface for *d* = 0.08 a.u. The quantum mechanical calculations have revealed that the conformation of the oligonucleotide strand of the aptaprobe is dependent on the temperature of the medium, ionic strength of the buffer electrolyte, and the concentration of Mg^2+^ ions. To determine the temperature ranges of the predominant conformations of ABP, the theoretical melting temperatures for the predominant conformations were calculated. They are listed together with the most stable ABP structures in Figure 1F. The calculations were carried out using UNAFold 3.9 program with Quikfold application provided at the DINAMelt web server of the University of Albany, New York [50,51]. The program is based on the unified base-pair theory. The obtained results indicate that the ABP can form four different small-loop hairpin structures, with melting temperatures from 37.1 °C for structure 1, 40.9 °C for structure 2, 43.4 °C for structure 3, to 49.6 °C for structure 4. These calculations corroborate the recent studies of the Myong group [52], indicating that the efficient formation of the G-quadruplex needs the help of ATP. Although the formation of a G-quadruplex is a thermodynamically favorable process for a random DNA strand, it requires some extensive bending of the DNA strand, and thus it is kinetically hindered. Hence, the faster formation of simpler, although metastable beacon forms, presented in Figure 1F, prevails in absence of other interacting species. However, these structures are metastable in the presence of ATP, and formation of the G-quadruplex is feasible due to the strong bonding of ATP to the guanines of ABP. It must be emphasized that aptamer structures 1–4, although predominant in temperature ranges up to 49.6 °C (see: Figure 1), are in thermodynamic equilibrium with the random coil structure of ABP, such as the one shown in Scheme 1, left panel. 

The fluorescence emission spectrum for 133 μM DNA aptaprobe in a TRIS-HCl buffer at room temperature of 21 °C is presented in Figure 1A. A well-defined emission peak at *λ* = 516 nm with intensity of 904.5 a.u. is observed. This peak is ascribed to the intrinsic fluorescence of FAM dye attached to the aptamer strand. As seen in Figure 1B, the peak emission intensity stabilizes within 15 min and then remains unchanged. Therefore, all subsequent experiments were performed after 15 min of ABP stabilization. At room temperature, the predominant conformation of the aptamer is represented by structure 1 in Figure 1F. In this structure, the distance between fluorophore FAM and quencher Dabcyl is large, and as such, no intra-molecular fluorescence quenching is expected. 

To evaluate the stability of ATP aptamer structure and its conformation changes, the influence of temperature on the operation of the aptamer beacon was investigated. As shown in Figure 1C, the temperature scan from 23 °C to 91 °C, with a scan rate of 2 deg/min, produced a reversible three-state (OFF-ON-OFF) melting characteristic of ABP. A similar phenomenon was observed for molecular beacon (MB) complementary to survivin mRNA [53]. It is seen that during the temperature scan from 23 °C to 67 °C, the intensity of the emission signal increases from F = 1 a.u. to F = 1.498 a.u. The distance between FAM dye at 5′ end of the aptamer and Dabcyl quencher at 3′ end increases, resulting in the increasing emission of the light by the fluorophore. Interestingly, upon further temperature increase from 67 °C to 91 °C, the fluorescence does not remain constant but decreases slightly to the level of F = 1.205 a.u. It becomes clear that with increasing temperature, the flexible single-stranded DNA aptamer assumes dynamically random conformations. The distance between FAM dye at the 5′ end of the aptamer and Dabcyl quencher at the 3′ end can momentarily come close enough to each other during the random arms’ waving to cause the self-quenching by the FRET process. The melting temperature of the highest-temperature stable conformation of ABP (structure 4) is *t*_m_ = 49.6 °C, which means that above this temperature, the concentration of conformation 4 is less than that of the random coil structures. The experimental apparent melting temperature *t*_m,app_, determined by the simplex fitting algorithm, is slightly higher than theoretically predicted for aptamer structure 4, and equals 51.4 ± 1.2 °C (Figure 1C). This value is slightly higher than that of structure 4, since conformations 1–3 are still present at temperatures above their melting points at low concentrations and contribute to the measured fluorescence emission. It is worth noting that for a shorter 27-nucleotide ATP-binding aptamer, originally developed by Huizenga and Szostak [39], the experimentally obtained melting temperature was 45.2 ± 0.5 °C, as determined by Slavkovic et al. [54] using UV melting curves. Therefore, the aptamer beacon probe developed in the present work shows somewhat higher stability at elevated temperatures than the benchmark probe of Huizenga and Szostak [39].

### 2.2. Temperature Dependence of the Interactions of Aptamer Beacon Probe with ATP and Other Nucleotides 

**Figure 2:**
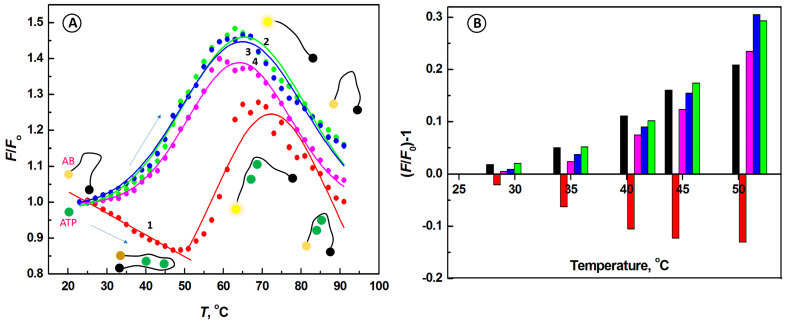
(**A**) Melting characteristics for the aptamer beacon probe with ATP (red, 1) and other nucleotides: GTP (green, 2), UTP (blue, 3), and CTP (violet, 4); INSET: pictorial view of changing conformations of aptaprobe alone and aptaprobe with ATP; arrows indicate direction of temperature scan. (**B**) The dependence of (*F*/*F*_o_)-1 vs. temperature for aptaprobe alone (black bar, 1), and aptaprobe interactions with nucleotides: remaining bars in each group: ATP (red, 2), CTP (violet, 3), UTP (blue, 4), and GTP (green, 5). Conditions: *C*_ABP_ = 133 nM, *C*_nucl_ = 625 µM; *λ*_ex_ = 480 nm, temperature scan: 23–91 °C; buffer solution: 20 mM Tris-HCl + 100 mM NaCl + 5 mM MgCl_2_.

In the presence of ATP, the temperature dependence of the aptaprobe fluorescence changes dramatically, as illustrated in Figure 2A, line 1. The fluorescence intensities were normalized according to the equation:

*I*_norm_ = *F*/*F*_o_ (1)

where *F* is the fluorescence intensity of aptamer in the presence of nucleotides, and *F*_o_ is the fluorescence intensity of ABP alone in buffer solution. 

During the temperature scan from 23 °C to 49 °C, performed after mixing of ATP with the aptamer beacon, the relative ABP fluorescence signal *F*/*F*_o_ decreased steadily from 1 to 0.867 (Figure 2, line 1, red), indicating the binding of ATP to the aptamer, concomitant with the aptamer undergoing conformation change. In the new ABP conformation, facilitating the aptamer beacon probe–ATP complex formation, the distance between the FAM dye and Dabcyl quencher becomes smaller, enabling the FRET process between them to occur. Hence, due to the high binding affinity of the aptamer to ATP, resulting in the enhanced FRET, the fluorescence intensity decreased despite the temperature increase, confirming the higher thermal stability of the ABP–ATP complex, in comparison to the bare aptamer conformations. During further temperature scan, above 51 °C, the fluorescence signal of ABP recovered, gradually increasing to reach the maximum level *F*/*F*_o_ = 1.247 at the peak temperature *t*_p_ = 69 °C, followed by a decrease to *F*/*F*_o_ = 1.02 at 91 °C due to the aptamer melting and formation of randomly coiled ABP structure. 

In further studies, the effects of other nucleotides, including UTP, GTP, and CTP, on the operation of the aptamer beacon were examined (Figure 2, lines 2–4). Unlike in the case of ATP (line 1, red), other nucleotides (GTP, line 2, green; UTP, line 3, blue; CTP, line 4, violet) do not cause remarkable changes in the aptamer probe melting characteristics after interactions of these nucleotides with the aptamer. It indicates, that in the cases of UTP, GTP, and CTP, the interactions of the aptamer with target nucleotides are much weaker in comparison to those with ATP, and no ABP-nucleotide complex is formed, leading to an unobstructed three-state transition, OFF-ON-OFF process, upon heating. Thus, the melting curves look similar to those for the bare aptamer (Figure 1C). These results confirm that the proposed aptamer probe binds specifically with ATP. We have observed his type of behavior, manifested by the increased quenching with increasing temperature, in other random coil DNA structures [53].

Figure 2B shows the dependence of the relative fluorescence change (*F*/*F*_o_)-1 on temperature for ABP interactions with ATP, UTP, GTP, and CTP. It is clearly seen that binding of the aptamer with ATP results in a fluorescence decrease, while for the aptaprobe alone and for aptaprobe binding with other nucleotides, the fluorescence signal increased. The decrease in fluorescence for aptamer beacon probe-ATP solutions was the largest for temperatures in the range from 40 °C to 50 °C, providing an excellent selectivity for ATP and best discrimination against other nucleotides. These findings indicate that the proposed aptamer probe offers a good selectivity and sensitivity.

### 2.3. Selectivity of the Biosensing System 

**Figure 3:**
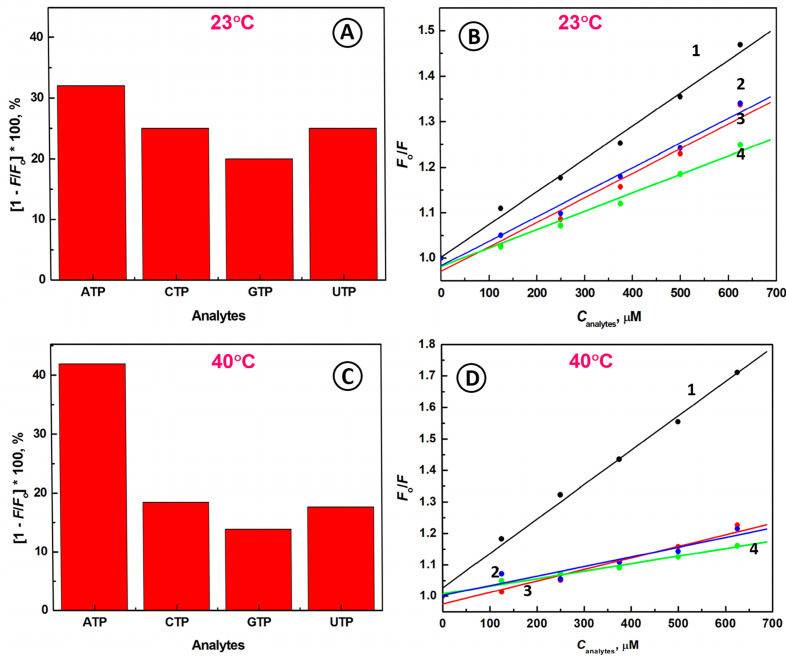
Fluorescence quenching efficiency of aptamer beacon after interaction with 625 µM solutions of nucleotides (ATP, CTP, GTP, UTP) at (**A**) 23 °C and (**C**) 40 °C; The Stern-Volmer plot of *F*_o_/*F* vs. *C*_nucl_: (1) ATP, (2) UTP, (3) CTP, (4) GTP, at (**B**) 23 °C and (**D**) 40 °C; Conditions: *C*_ABP_ = 133 nM, *λ*_ex_ = 480 nm; solution: 20 mM Tris-HCl buffer + 100 mM NaCl + 5 mM MgCl_2_.

Testing of the selectivity of aptamer beacon probe was performed with triphosphate nucleotides, as illustrated in Figure 3. It is clearly seen that the interactions of aptaprobe with ATP at 23 °C induced stronger quenching of fluorescence than those of ABP with other nucleotides (Figure 3A,B). The observed aptaprobe fluorescence quenching efficiency (QE) for ATP was QE = 32%, while that for other nucleotides was 25%, 25%, and 20%, for CTP, UTP, and GTP, respectively. It indicates a higher affinity of the aptamer for ATP in comparison with that of other nucleotides. However, the difference between QE values for ATP and the remaining nucleotides at 23 °C was insufficient for discrimination against the effects of other nucleotides and effective ATP determination. To gain more insight into the nature of the binding of the aptaprobe with nucleotides and to optimize conditions for the expansion of the gap between QE for ATP and other nucleotides, the Stern-Volmer quenching dependencies were investigated. As such, we have determined the Stern-Volmer quenching constants *K*_SV_ for ATP, UTP, GTP, and CTP. As shown in Figure 3B, to determine the quenching constant *K*_SV_, the values of the ratio *F*_o_/*F* were plotted against the concentration of different nucleotides *C*_nucl_. According to the Stern-Volmer equation, we have:

*F_o_*/*F* = 1 + *K*_SV_*C*_nucl_ (2)

where *F*_o_ and *F* are the fluorescence intensities for the aptamer beacon probe in the absence and the presence of nucleotides, respectively; *K*_SV_ is the quenching constant, and *C*_nucl_ is the concentration of the respective nucleotide—ATP, CTP, UPT, or GTP—acting as the quencher. 

The fluorescence quenching data obtained for 23 °C have revealed that the Stern-Volmer quenching constants are *K*_SV_ = 721 ± 31 M^−1^ for ATP, *K*_SV_ = 540 ± 49 M^−1^ for CTP, *K*_SV_ = 540 ± 33 M^−1^ for UTP, and *K*_SV_ = 405 ± 28 M^−1^ for GTP. These results confirm the highest affinity of the aptamer beacon probe towards ATP molecules.

Due to the largest fluorescence signal decrease for interaction of ABP with ATP in the temperature range of 40 °C to 50 °C, a temperature of 40 °C was chosen for subsequent experiments. At 40 °C, the fluorescence quenching efficiency (QE) of aptaprobe towards ATP was significantly higher in comparison to that of room temperature, and the value of QE = 42% was obtained (Figure 3C). In contrast, for other nucleotides such as UTP, GTP, and CTP, much lower values of QEs were achieved at 40 °C, approaching QE = 18% for CTP, QE = 14% for GTP, and QE = 18% for UTP. Hence, by optimizing the experimental conditions, we were able to increase the response of the ABP towards ATP, while at the same time, the ABP response towards other nucleotides decreased significantly.

In order to see how Stern-Volmer quenching constants and the quenching process are affected by temperature, the interactions of aptaprobe with ATP and other nucleotides including UTP, GTP, and CTP have been investigated at 40 °C (Figure 3D). We have found that the value of the Stern-Volmer quenching constant for ATP (Figure 3D, line 1) increased 1.52-fold to *K*_SV_ = 1093 ± 43 M^−1^, in comparison to the value of *K*_SV_ obtained at room temperature (*K*_SV_ = 721 ± 31 M^−1^). These results clearly indicate that a stronger binding affinity of ATP to the aptamer beacon probe is observed at higher temperature. The increase of *K*_SV_ with temperature is likely to be due to the activation of the aptamer conformational transformation, enabling stronger ATP binding. On the other hand, the values of the Stern-Volmer quenching constants for UTP, GTP, and CTP nucleotides decreased at 40 °C to *K*_SV_ = 367 ± 42 M^−1^ for CTP (Figure 3D, line 2), *K*_SV_ = 307 ± 45 M^−1^ for UTP (Figure 3D, line 3), and 240 ± 15 M^−1^ for GTP (Figure 3D, line 4), respectively. These results additionally confirm the high specificity of aptaprobe toward ATP in comparison with UTP, CTP, and GTP nucleotides. 

These results corroborate the data obtained in earlier experiments, and indicate that aptamer beacon probe is specific and suitable for the detection of ATP.

### 2.4. Detection of ATP Molecules

**Figure 4:**
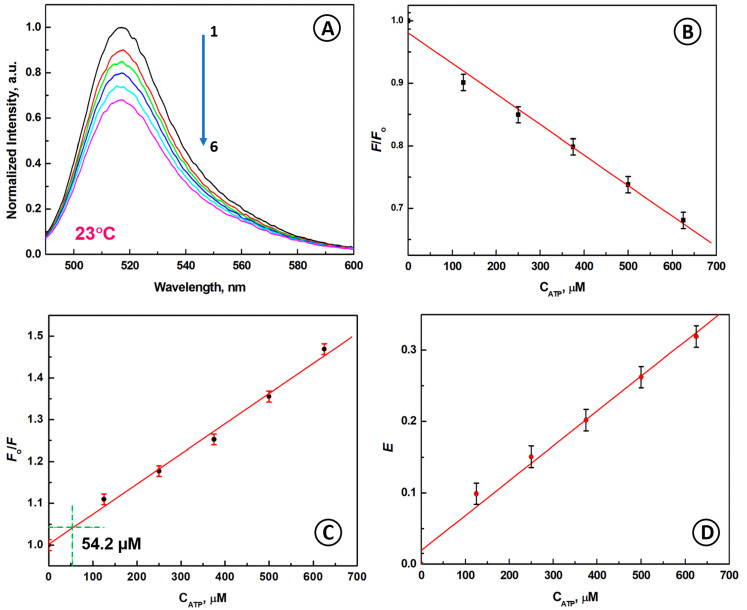
(**A**) Normalized fluorescence emission spectra of aptamer beacon for different concentrations of ATP; (**B**) The dependence of *F/F*_o_ vs. C_ATP_; (**C**) The dependence of *F*_o_/*F* vs. *C*_ATP_ showing the 3σ LOD with a dashed line; (**D**) Dependence of quenching efficiency *E* on *C*_ATP_; conditions: *C*_ATP_ [µM]: (1) 0, (2) 125, (3) 250, (4) 375, (5) 500, (6) 625; *C*_ABP_ = 133 nM, *λ*_ex_ = 480 nm; solution: 20 mM Tris-HCl buffer + 100 mM NaCl + 5 mM MgCl_2_, temperature 23 °C.

The feasibility of the determination of ATP using the developed aptamer beacon probe was investigated using melting temperature measurements and fluorescence spectroscopy. As illustrated in Figure 4A, the interactions of ATP molecules with the aptamer beacon probe resulted in quenching of aptamer FAM fluorescence by 32% for 625 µM ATP, at 23 °C. 

The dependence of *F/F*_o_ vs. *C*_ATP_ is presented in Figure 4B. Upon the addition of ATP to the sensing system, a fluorescence change that is inversely proportional to the ATP concentration increase from 0 to 625 µM was observed. The Stern-Volmer plot is presented in Figure 4C. The linear fitting was performed for the obtained experimental data, obtaining the following calibration line equation:

*F*_o_/*F* = 1 + 0.72 × 10^−3^ *C*_ATP_ (3)

where *C*_ATP_ is expressed in [μM]. The LOD, determined for the standard 3σ deviation is:

LOD = 3σ/slope = 3 × 0.013/0.00072 = 54.2 µM(4)

The quenching efficiency (0–0.32) of the aptamer beacon for different concentrations of ATP is shown in Figure 4D. It is seen that at 23 °C, the plot of quenching efficiency *E* vs. *C*_ATP_ is linear in the investigated ATP concentration range.

**Figure 5:**
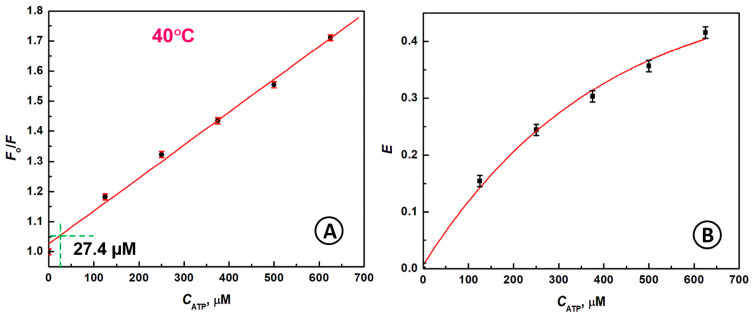
(**A**) The dependence of *F*_o_/*F* vs. *C*_ATP_ showing the 3σ LOD with a dashed line; (**B**) Dependence of quenching efficiency *E* on *C*_ATP_; conditions: *C*_ATP_ [µM]: (1) 0, (2) 125, (3) 250, (4) 375, (5) 500, (6) 625; *C*_ABP_ = 133 nM, *λ*_ex_ = 480 nm; solution: 20 mM Tris-HCl buffer + 100 mM NaCl + 5 mM MgCl_2_, temperature 40 °C.

By increasing the temperature to 40 °C, the ATP detection limit can be further decreased to LOD = 27.4 µM ATP, as is shown in Figure 5A. In Figure 5B, the dependence of *E* vs. *C*_ATP_ is depicted. Obviously, the higher temperature causes much greater fluorescence self-quenching (42%) of the ABP–ATP complex than that observed at a lower temperature (31%), for the same 625 µM ATP concentration.

A comparison of the aptamer sequences, conditions, and detection limits reported recently for ATP sensors and assays based on DNA and RNA aptamers is presented in Table 1. The sensitivity and operational simplicity of the fluorescence turn-off aptaprobe biosensing system developed in this work are competitive to other biosensing assays listed in this table. 

### 2.5. Determination of ATP in Cell Lysate Matrix Using Standard Addition Procedure

In the next step towards monitoring ATP levels in cancer cells, the applicability of the proposed biosensing assay was tested in the presence of SW480 cancer cell lysate, diluted 300-fold in buffer solution. The lysate of cancer cells treated with 10 mM 2-deoxyglucose (2-DG) for 24 h was also used in testing. The concentration of ATP in lysate solutions was determined using a new method derived from the basic Stern-Volmer Equation (2). This method enables simultaneous determination of the *K*_SV_ quenching constant in the presence of the lysate matrix and the ATP concentration in the lysate. For this purpose, the Stern-Volmer equation has been rewritten in the form:

*F*_o_*/F =* 1 *+ K*_SV_*C*_res_ + *K*_SV_*C*_added_ (5)

where *C*_res_ is the unknown concentrations of ATP introduced to the sample from lysate, and *C*_added_ is the concentrations of ATP added in the standard addition procedure. In the series of solutions analyzed, the term 1 + *K*_SV_*C*_res_ remains constant, so we define it as:

*K*_res_ = 1 + *K*_SV_*C*_res_ (6)

The new form of the Stern-Volmer equation:

*F*_o_*/F = K*_res_ + *K*_SV_*C*_added_ (7)

indicates that the graphs of the Stern-Volmer function *F*_o_*/F* plotted against the added quencher concentration *C*_added_ will no longer pass through the terminal point 0,1, which is now replaced with a new critical point 0, *K*_res_. This new characteristic point is critical since it is dependent on the concentration of the “hidden” quencher (ATP) in the lysate and enables its determination.

**Figure 6:**
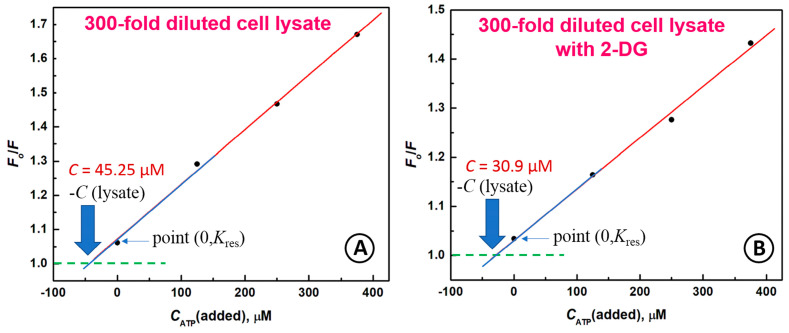
(**A**) The standard addition procedure for detection of ATP concentrations in cell lysate (n = 3) (**A**) and lysate of cells treated with 10 mM 2-DG for 24 h (n = 2); (**B**) Conditions: *C*_ABP_ = 133 nM, *C*_ATP_ [µM]: (1) 0, (2) 125, (3) 250, (4) 375; *λ*_ex_ = 480 nm; solution: SW480 cells lysate 300-fold diluted in 20 mM Tris-HCl buffer with 100 mM NaCl + 5 mM MgCl_2_.

This determination can be performed either graphically, as illustrated in Figure 6, or by calculation using the formula:

*C*_res_ = (*K*_res_ − 1)/*K*_SV_ (8)

The solution from cell lysate (300-fold diluted in buffer solution) was spiked with an ATP solution to obtain increasing ATP concentrations: 125 µM, 250 µM, and 375 µM. The experiments were repeated triply for untreated cells and twice for treated cells. It was found that the obtained relative standard deviation (RSD) for ATP determination was between 5.87% and 9.88 % for untreated SW480 cell lysate, and between 3.86% and 4.17% for lysate from cell treated with 2-DG (Table 2). The obtained results indicate that the inhibition of glycolysis in SW480 cancer cells by 10 mM 2-deoxyglucose (2-DG) for 24 h results in lowering of ATP production by 31.7%. Similar results were obtained by Choi and Lee [60]. The authors have shown ca. 20% ATP depletion in HeLa cells treated with 10 mM concentration of 2-DG for 24 h. Zhao et al. [61] observed a decrease of ATP synthesis by ca. 25% after treatment of WT 9-7 cells with 5 mM 2-DG for 48 h.

This means that the effective control of the excess energy production, necessary for cancer progression, can be achieved by suppressing the intracellular ATP level, enabling the inhibition of the tumor growth and metastasis. 

In our previous studies, we have utilized an “On-Off” switching DNA aptamer for transfection of the SW480 cancer cells, using Lipofectamine carriers, for monitoring of ATP levels in untreated and oligomycin-treated SW480 cells [49]. The images of fluorescence emission from the aptamer probe in transfected cells were successfully acquired with a Nikon Eclipse TE300 inverted light microscope with fluorescence filters. Therefore, the aptasensor developed in the present work has potential for future utilization in imaging of ATP in cancer cells. It can also aid in the development of new methods of energy production modulation, enabling the inhibition of cancer growth and metastasis. Hence, the proposed biosensing assay may enable the development of new anticancer therapies based on reprogramming the energy production in cancer cells.

## 3. Materials and Methods

### 3.1. Chemicals

The novel DNA aptamer beacon probe, designed for recognition of ATP, consisted of a 37-mer oligonucleotide strand and a fluorophore-quencher pair (FAM-Dabcyl) attached to the ends of the strand with the following sequence: 5′-FAM-ATTCTCTCACCTGGGGGAGTATTGCGGAGGAAGGTAT-Dabcyl-3′. It was synthesized by the FUTUREsynthesis Sp. z o.o. Poznan, Poland. The purity of this oligonucleotide was tested with HPLC. Adenosine 5′-triphosphate disodium salt hydrate (ATP), guanosine 5′-triphosphate sodium salt hydrate (GTP), cytidine 5′-triphosphate disodium salt (CTP), uridine 5′-triphosphate trisodium salt dihydrate (UTP), trizma hydrochloride (Tris-HCl), magnesium chloride (MgCl_2_), sodium chloride (NaCl), and 2-deoxyglucose (2-DG) were obtained from Sigma-Aldrich Chemical Company (St. Louis, MO, USA). All chemicals were of analytical grade purity. Aqueous solutions were prepared with freshly deionized water with 18.2 MΩ cm resistivity (Millipore, Poland). All concentrations of added reagents cited in this paper are final concentrations obtained after mixing, unless otherwise noted.

### 3.2. Instrumentation

The fluorescence spectra were recorded using Spectrometer model LS55 (Perkin Elmer, Waltham, MA, USA), with a 20 kW pulsed Xenon light source and a photomultiplier tube detector. The excitation and emission slit widths were set to 5.0 nm and scan speed to 500 nm/min. The excitation wavelength was set to *λ*_ex_ = 480 nm. The measurements were performed in 20 mM Tris-HCl buffer + 100 mM NaCl + 5 mM MgCl_2_ solutions with pH 7.4. During the temperature dependence experiments, the temperature was scanned stepwise with a step height of 2 °C, and measurements were performed after 1 min of waiting at each temperature.

### 3.3. Cell Culture

The experiments with cancer cells were performed using a SW480 cell line purchased from ATCC (LGC Standards Sp. z.o.o., Lomianki, Poland). The cells were cultured in DMEM (Dulbecco’s modified Eagle’s Medium) with added 10% FBS (fetal bovine serum, Gibco, Rockville, MD, USA) and were maintained in a humidified air atmosphere containing 5% CO_2_ at 37 °C (Shel Lab Model 2123-TC CO_2_ Incubator Cornelius, OR, USA). Every 3 days, the cells were further subcultured. The cells remaining after the experiments were handled according to safety protocols.

### 3.4. Cell Lysate

SW480 cells were detached from the culture dish by 2 mL trypsin-EDTA at 37 °C for 5–10 min and centrifuged twice in PBS solution at 0.9 RPM for 3.5 min to pellet the cells. Then, 1500 μL of a lysis buffer containing dimethyl sulfoxide and ethanol (DMSO:EtOH = 1:1) was added to the cells’ pellet and centrifuged at 4 °C for 10 min at 14,000 *g*. The supernatant obtained was 10 times diluted with Tris-HCl buffer and used as a stock solution of the cell lysate in the following experiments. Next, 100 μL of cell lysate with final dilution 1:300 was added to the measurement buffer (20 mM Tris-HCl, 100 mM NaCl, 5 mM MgCl_2_) with ABP. Then, solutions with different concentrations of ATP (125, 250, and 375 μM ATP, final concentration) were added and fluorescence measurements, at the excitation wavelength *λ*_ex_ = 480 nm, were performed.

In experiments with glycolysis inhibitors, the SW480 cell line culture was first exposed to 10 mM 2-DG for 24 h, followed by the cells’ lysis.

### 3.5. Calculations

The calculations of predominant aptaprobe structures and their melting temperatures were performed using the University of Albany web server DINAMelt, providing the program UNAFold ver. 3.9 with a Quikfold application (RNA Institute, University of Albany, Albany, NY, USA).

The curve fitting to the experimental data points was carried out using the simplex routine embedded in the Origin graphing software (OriginLab Corp., Northampton, MA, USA, https://www.originlab.com/). 

## 4. Conclusions

In this work, we have developed a rapid and highly sensitive aptamer beacon probe for fluorescent detection of ATP in cancer cell lysate. The proposed aptamer beacon probe is based on the single-stranded oligonucleotide specific to ATP molecules, and is functionalized with a fluorescent dye (FAM) and a quencher (Dabcyl) at the opposite ends of the probe strand. The likely interferents, such as molecules with similar chemical structure, including GTP, CTP, and UTP, have also been investigated. Several experimental parameters, including the incubation time of ABP and the effect of experimental temperature on the biosensing assay performance, were investigated using quenching efficiency (QE) and Stern-Volmer constants (*K*_SV_). We have found that the best selectivity of the aptamer beacon probe toward ATP was observed at 40 °C, for which the parameters *K*_SV_ = 1093 M^−1^ and QE = 42% were obtained. The proposed biosensing assay enables the detection of ATP concentration in a 300-fold diluted cells’ lysate, obtained from both the untreated SW480 cancer cells, as well as from cells treated with 2-deoxyglucose (2-DG). We have shown that the applied treatment with 2-DG glycolysis inhibitor results in lowering of ATP production by 31.7%. The obtained results indicate that ATP may act as a non-specific cancer biomarker in a multi-panel platform for cancer diagnostics and screening. We have demonstrated that the proposed aptamer beacon probe for fluorometric monitoring of ATP level modulation can serve as a powerful tool in future cancer treatments based on controlling energy sources in cancer cells and the tumor microenvironment. Since ATP is also formed in many processes other than the cellular respiration, such as in the beta-oxidation, ketosis, lipid- and protein-catabolism, as well as under anaerobic conditions [62], further investigations of slowing down ATP production in cancer cells, which is required for their extensive energy needs for proliferation and metastasis, are warranted.

## Figures and Tables

**Table 1:** Examples of ATP detection using fluorescence turn-on aptamer beacon probes.

No.	Fluorescence Aptaprobe	*C*_probe_, µM	Buffer Solution	ATP Detection (LOD), [µM]	References
1.	Cy5-TGGAAGGAGGCG TTA TGA GGG GGT CCA	3.3	300 mM NaCl, 20 mM Tris-HCl, 0.1% Tween 20, pH = 8.3	34	[55]
2.	DNA1-GGGTTGGG/iSp9/ACCTGGGGGAGTA	12	Tris-HCl, pH 9.0	28	[56]
	DNA2-CCCTGCGGAGGAAGGT/iSp9/GGGTAGGG	4			
3.	TCTCTCACCTGGGGGAGTATTGCGGAGGAAGGT-FAM	0.0667	20 mM Tris-HCl, 100 mM NaCl, 5 mM MgCl_2_, pH 7.4	17	[49]
4.	FAM-ATTCTCTCACCTGGGGGAGTATTGCGGAGGAAGGTAT-Dabcyl	0.133	20 mM Tris-HCl buffer, 100 mM NaCl 5 mM MgCl_2_, pH 7.4	27	This work
5.	ATACCTGGGGAGTATAGCTGTGTGACGCAACACTAGCGGAGGAAGGTAT	0.5	25 mM Tris-HCl, 120 mM NaCl, 5 mM KCl, 3 mM MgCl_2_, 0.02% Tween20, pH 7.4	40	[57]
6.	GUCGCACGUAGUGG-PyUGUGUGUGUGGUGCGAC	1.5	10 mM Tris-HCl buffer, pH 7.2, 100 mM NaCl, 10 mM MgCl_2_	90	[58]
7.	GUCGCACGPyUAGUGG-UGUGUGUGUGGUGCGAC	1.5	10 mM Tris-HCl buffer, pH 7.2, 100 mM NaCl, 10 mM MgCl_2_	820	[58]
8.	FAM-ACCTGGGGGAGTATTGCGGAGGAAGGT T_10_	0.5	15 mM Tris buffer, pH 8.5	28	[59]
9.	FAM-ACC TGG GGG AGT ATT GCG GAG GAA GGT-NH_2_	20	15 mM Tris buffer, pH 8.5	19	[59]

**Table 2:** The relative standard deviation (RSD) for ATP in 300-fold diluted cell lysates (n = 2–3).

Samples	Added, µM	RSD (%)
lysate from untreated SW480 cancer cells	125	5.87
	250	9.01
	375	9.88
lysate from SW480 cancer cells treated with 2-DG	125	4.17
	250	3.86
	375	4.15

## Data Availability

Not applicable.

## References

[1] Sung H., Ferlay J., Siegel R.L., Laversanne M., Soerjomataram I., Jemal A., Bray F. (2021). Global Cancer Statistics 2020: GLOBOCAN Estimates of Incidence and Mortality Worldwide for 36 Cancers in 185 Countries. CA Cancer J. Clin..

[2] Xi Y., Xu P. (2021). Global colorectal cancer burden in 2020 and projections to 2040. Transl. Oncol..

[3] Siegel R.L., Wagle N.S., Cercek A., Smith R.A., Jemal A. (2023). Colorectal cancer statistics, 2023. CA Cancer J. Clin..

[4] Stobiecka M., Ratajczak K., Jakiela S. (2019). Toward early cancer detection: Focus on biosensing systems and biosensors for an anti-apoptotic protein survivin and survivin mRNA. Biosens. Bioelectron..

[5] Ratajczak K., Krazinski B.E., Kowalczyk A.E., Dworakowska B., Jakiela S., Stobiecka M. (2018). Hairpin–Hairpin Molecular Beacon Interactions for Detection of Survivin mRNA in Malignant SW480 Cells. ACS Appl. Mater. Interfaces.

[6] Ratajczak K., Krazinski B.E., Kowalczyk A.E., Dworakowska B., Jakiela S., Stobiecka M. (2018). Optical Biosensing System for the Detection of Survivin mRNA in Colorectal Cancer Cells Using a Graphene Oxide Carrier-Bound Oligonucleotide Molecular Beacon. Nanomaterials.

[7] Stobiecka M., Dworakowska B., Jakiela S., Lukasiak A., Chalupa A., Zembrzycki K. (2016). Sensing of Survivin mRNA in Malignant Astrocytes Using Graphene Oxide Nanocarrier-Supported Oligonucleotide Molecular Beacons. Sens. Actuat. B.

[8] Stobiecka M., Chalupa A., Dworakowska B. (2016). Piezometric biosensors for anti-apoptotic protein survivin based on buried positive-potential barrier and immobilized monoclonal antibodies. Biosens. Bioelectron..

[9] Ratajczak K., Stobiecka M. (2017). Ternary Interactions and Energy Transfer Between Fluorescein Isothiocyanate, Adenosine Triphosphate, and Graphene Oxide Nanocarriers. J. Phys. Chem. B.

[10] Coppi E., Pugliese A.M., Urbani S., Melani A., Cerbai E., Mazzanti B., Bosi A., Saccardi R., Pedata F. (2007). ATP Modulates Cell Proliferation and Elicits Two Different Electrophysiological Responses in Human Mesenchymal Stem Cells. Stem Cells.

[11] Rajendran M., Dane E., Conley J., Tantama M. (2016). Imaging Adenosine Triphosphate (ATP). Biol. Bull..

[12] Rigoulet M., Bouchez C.L., Paumard P., Ransac S., Cuvellier S., Duvezin-Caubet S., Mazat J.P., Devin A. (2020). Cell energy metabolism: An update. Biochim. Biophys. Acta (BBA) Bioenerg..

[13] Martinello T., Baldoin M.C., Morbiato L., Paganin M., Tarricone E., Schiavo G., Bianchini E., Sandonà D., Betto R. (2011). Extracellular ATP signaling during differentiation of C2C12 skeletal muscle cells: Role in proliferation. Mol. Cell. Biochem..

[14] Salvestrini V., Orecchioni S., Talarico G., Reggiani F., Mazzetti C., Bertolini F., Orioli E., Adinolfi E., Di Virgilio F., Pezzi A. (2017). Extracellular ATP induces apoptosis through P2X7R activation in acute myeloid leukemia cells but not in normal hematopoietic stem cells. Oncotarget.

[15] Fiorillo M., Ózsvári B., Sotgia F., Lisanti M.P. (2021). High ATP Production Fuels Cancer Drug Resistance and Metastasis: Implications for Mitochondrial ATP Depletion Therapy. Front. Oncol..

[16] Di Virgilio F., Sarti A.C., Falzoni S., De Marchi E., Adinolfi E. (2018). Extracellular ATP and P2 purinergic signalling in the tumour microenvironment. Nat. Rev. Cancer.

[17] Vultaggio-Poma V., Sarti A.C., Di Virgilio F. (2020). Extracellular ATP: A Feasible Target for Cancer Therapy. Cells.

[18] Di Virgilio F., Adinolfi E. (2017). Extracellular purines, purinergic receptors and tumor growth. Oncogene.

[19] Yang H., Geng Y.-H., Wang P., Zhou Y.-T., Yang H., Huo Y.-F., Zhang H.-Q., Li Y., He H.-Y., Tian X.-X. (2019). Extracellular ATP promotes breast cancer invasion and epithelial-mesenchymal transition via hypoxia-inducible factor 2α signaling. Cancer Sci..

[20] Yang H., Geng Y.-H., Wang P., Zhang H.-Q., Fang W.-G., Tian X.-X. (2022). Extracellular ATP promotes breast cancer chemoresistance via HIF-1α signaling. Cell Death Dis..

[21] Fiorillo M., Scatena C., Naccarato A.G., Sotgia F., Lisanti M.P. (2021). Bedaquiline, an FDA-approved drug, inhibits mitochondrial ATP production and metastasis in vivo, by targeting the gamma subunit (ATP5F1C) of the ATP synthase. Cell Death Differ..

[22] Zhou Y., Tozzi F., Chen J., Fan F., Xia L., Wang J., Gao G., Zhang A., Xia X., Brasher H. (2012). Intracellular ATP levels are a pivotal determinant of chemoresistance in colon cancer cells. Cancer Res..

[23] Hanahan D., Weinberg R.A. (2011). Hallmarks of Cancer: The Next Generation. Cell.

[24] Pelicano H., Martin D., Xu R.-H., Huang P. (2006). Glycolysis inhibition for anticancer treatment. Oncogene.

[25] Zhao Y., Butler E., Tan M. (2013). Targeting cellular metabolism to improve cancer therapeutics. Cell Death Dis..

[26] Martin D.S., Bertino J.R., Koutcher J.A. (2000). ATP Depletion + Pyrimidine Depletion Can Markedly Enhance Cancer Therapy: Fresh Insight for a New Approach1. Cancer Res..

[27] Martin D.S., Stolfi R.L., Colofiore J.R., Nord L.D., Sternberg S. (1994). Biochemical Modulation of Tumor Cell Energy in Vivo: II. A Lower Dose of Adriamycin is Required and a Greater Antitumor Activity is Induced when Cellular Energy is Depressed. Cancer Investig..

[28] Stolfi R.L., Colofiore J.R., Nord L.D., Martin D.S. (1996). Enhanced antitumor activity of an adriamycin + 5-fluorouracil combination when preceded by biochemical modulation. Anti-Cancer Drugs.

[29] Stolfi R.L., Colofiore J.R., Nord L.D., Koutcher J.A., Martin D.S. (1992). Biochemical Modulation of Tumor Cell Energy: Regression of Advanced Spontaneous Murine Breast Tumors with a 5-Fluorouracil-containing Drug Combination1. Cancer Res..

[30] Wang H., Li Y., Zhang M., Wu D., Shen Y., Tang G., Ping Y. (2017). Redox-Activatable ATP-Depleting Micelles with Dual Modulation Characteristics for Multidrug-Resistant Cancer Therapy. Adv. Healthc. Mater..

[31] Song X.-R., Li S.-H., Guo H., You W., Tu D., Li J., Lu C.-H., Yang H.-H., Chen X. (2018). Enhancing Antitumor Efficacy by Simultaneous ATP-Responsive Chemodrug Release and Cancer Cell Sensitization Based on a Smart Nanoagent. Adv. Sci..

[32] Kaushik N., Lee S.J., Choi T.G., Baik K.Y., Uhm H.S., Kim C.H., Kaushik N.K., Choi E.H. (2015). Non-thermal plasma with 2-deoxy-D-glucose synergistically induces cell death by targeting glycolysis in blood cancer cells. Sci. Rep..

[33] Chow L.W.C., Cheng K.-S., Leong F., Cheung C.-W., Shiao L.-R., Leung Y.-M., Wong K.-L. (2019). Enhancing tetrandrine cytotoxicity in human lung carcinoma A549 cells by suppressing mitochondrial ATP production. Naunyn-Schmiedeberg’s Arch. Pharmacol..

[34] Maximchik P., Abdrakhmanov A., Inozemtseva E., Tyurin-Kuzmin P.A., Zhivotovsky B., Gogvadze V. (2018). 2-Deoxy-D-glucose has distinct and cell line-specific effects on the survival of different cancer cells upon antitumor drug treatment. FEBS J..

[35] Ben Sahra I., Laurent K., Giuliano S., Larbret F., Ponzio G., Gounon P., Le Marchand-Brustel Y., Giorgetti-Peraldi S., Cormont M., Bertolotto C. (2010). Targeting cancer cell metabolism: The combination of metformin and 2-deoxyglucose induces p53-dependent apoptosis in prostate cancer cells. Cancer Res..

[36] Javaherian S., Musheev M.U., Kanoatov M., Berezovski M.V., Krylov S.N. (2009). Selection of aptamers for a protein target in cell lysate and their application to protein purification. Nucleic Acids Res..

[37] Aljohani M.M., Cialla-May D., Popp J., Chinnappan R., Al-Kattan K., Zourob M. (2022). Aptamers: Potential Diagnostic and Therapeutic Agents for Blood Diseases. Molecules.

[38] Grabowska I., Sharma N., Vasilescu A., Iancu M., Badea G., Boukherroub R., Ogale S., Szunerits S. (2018). Electrochemical Aptamer-Based Biosensors for the Detection of Cardiac Biomarkers. ACS Omega.

[39] Huizenga D.E., Szostak J.W. (1995). A DNA aptamer that binds adenosine and ATP. Biochemistry.

[40] Zhang Z., Oni O., Liu J. (2017). New insights into a classic aptamer: Binding sites, cooperativity and more sensitive adenosine detection. Nucleic Acids Res..

[41] Biniuri Y., Luo G.-F., Fadeev M., Wulf V., Willner I. (2019). Redox-Switchable Binding Properties of the ATP–Aptamer. J. Am. Chem. Soc..

[42] Ng S., Lim H.S., Ma Q., Gao Z. (2016). Optical Aptasensors for Adenosine Triphosphate. Theranostics.

[43] Liu W., Zhu X., Mozneb M., Nagahara L., Hu T.Y., Li C.-Z. (2021). Lighting up ATP in cells and tissues using a simple aptamer-based fluorescent probe. Microchim. Acta.

[44] Guo Y., Wu J., Ju H. (2015). Target-driven DNA association to initiate cyclic assembly of hairpins for biosensing and logic gate operation. Chem. Sci..

[45] Zhou C., Yu Z., Yu W., Liu H., Zhang H., Guo C. (2019). Split aptamer-based detection of adenosine triphosphate using surface enhanced Raman spectroscopy and two kinds of gold nanoparticles. Microchim. Acta.

[46] Wu Y., Wang C., Wang C., Wang P., Chang X., Han L., Zhang Y. (2022). Multiple Biomarker Simultaneous Detection in Serum via a Nanomaterial-Functionalized Biosensor for Ovarian Tumor/Cancer Diagnosis. Micromachines.

[47] Wang Y., Luo J., Liu J., Sun S., Xiong Y., Ma Y., Yan S., Yang Y., Yin H., Cai X. (2019). Label-free microfluidic paper-based electrochemical aptasensor for ultrasensitive and simultaneous multiplexed detection of cancer biomarkers. Biosens. Bioelectron..

[48] Kuntamung K., Jakmunee J., Ounnunkad K. (2021). A label-free multiplex electrochemical biosensor for the detection of three breast cancer biomarker proteins employing dye/metal ion-loaded and antibody-conjugated polyethyleneimine-gold nanoparticles. J. Mater. Chem. B.

[49] Ratajczak K., Lukasiak A., Grel H., Dworakowska B., Jakiela S., Stobiecka M. (2019). Monitoring of dynamic ATP level changes by oligomycin-modulated ATP synthase inhibition in SW480 cancer cells using fluorescent “On-Off” switching DNA aptamer. Anal. Bioanal. Chem..

[50] Markham N.R., Zuker M. (2005). DINAMelt Web Server for Nucleic Acid Melting Prediction. Nucleic Acids Res..

[51] Markham N., Zuker M., Keith J.M. (2008). UNAFold: Software for Nucleic Acid Folding and Hybridization. Bioinformatics: Structure, Function and Applications.

[52] Tippana R., Chen M.C., Demeshkina N.A., Ferré-D’Amaré A.R., Myong S. (2019). RNA G-quadruplex is resolved by repetitive and ATP-dependent mechanism of DHX36. Nat. Commun..

[53] Stobiecka M., Chalupa A. (2016). DNA Strand Replacement Mechanism in Molecular Beacons Encoded for Detection of Cancer Biomarkers. J. Phys. Chem. B.

[54] Slavkovic S., Zhu Y., Churcher Z.R., Shoara A.A., Johnson A.E., Johnson P.E. (2020). Thermodynamic analysis of cooperative ligand binding by the ATP-binding DNA aptamer indicates a population-shift binding mechanism. Sci. Rep..

[55] Wang Y., Wang Y., Liu B. (2008). Fluorescent detection of ATP based on signaling DNA aptamer attached silica nanoparticles. Nanotechnology.

[56] Wang D., Geng F., Wang Y., Ma Y., Li G., Qu P., Shao C., Xu M. (2020). Design of a Fluorescence Turn-on and Label-free Aptasensor Using the Intrinsic Quenching Power of G-Quadruplex to AMT. Anal. Sci. Int. J. Jpn. Soc. Anal. Chem..

[57] Bing T., Mei H., Zhang N., Qi C., Liu X., Shangguan D. (2014). Exact tailoring of an ATP controlled streptavidin binding aptamer. RSC Adv..

[58] Park Y., Nim-anussornkul D., Vilaivan T., Morii T., Kim B.H. (2018). Facile conversion of ATP-binding RNA aptamer to quencher-free molecular aptamer beacon. Bioorganic Med. Chem. Lett..

[59] Liu Z., Chen S., Liu B., Wu J., Zhou Y., He L., Ding J., Liu J. (2014). Intracellular Detection of ATP Using an Aptamer Beacon Covalently Linked to Graphene Oxide Resisting Nonspecific Probe Displacement. Anal. Chem..

[60] Choi Y., Lee J.H. (2011). The combination of tephrosin with 2-deoxy-D-glucose enhances the cytotoxicity via accelerating ATP depletion and blunting autophagy in human cancer cells. Cancer Biol. Ther..

[61] Zhao J., Ma Y., Zhang Y., Fu B., Wu X., Li Q., Cai G., Chen X., Bai X.-Y. (2019). Low-dose 2-deoxyglucose and metformin synergically inhibit proliferation of human polycystic kidney cells by modulating glucose metabolism. Cell Death Discov..

[62] Dunn J., Grider M.H. (2023). Physiology, Adenosine Triphosphate. StatPearls.

